# Supplementary material for: Enhanced Anti-Inflammatory Activity of Tilianin Based on the Novel Amorphous Nanocrystals
Source: Pharmaceuticals (Basel). 2024 May 17;17(5):654. doi: 10.3390/ph17050654 (PMC11125044; doi:10.3390/ph17050654)
Supplement: Supplementary file 1 [file pharmaceuticals-17-00654-s001.zip › pharmaceuticals-2974214-supplementary.pdf]

Supporting information

1. Results and discussion

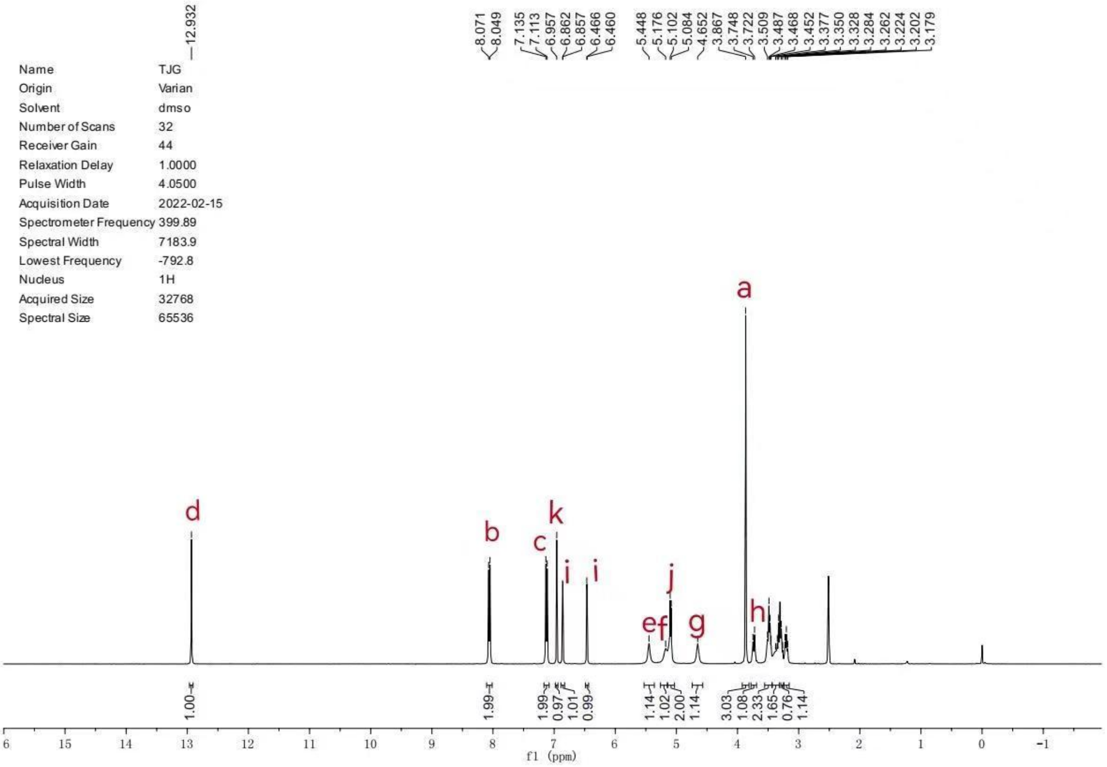

Figure S1. The H^{1}NMR detection profile of tilianin.

In H^{1}NMR spectra (Figure S1), a denotes H on the methyl group, b, c, k and i represent hydrogen atoms from the benzene ring, d denotes H from phenolic hydroxyl group. The e, f, g, h and j represent H from the sugar group. The specific peak position and integration proves the chemical structure of Til.
